# Ischemic Stroke Presenting as the First Symptom in a Setting of Paroxysmal Nocturnal Hemoglobinuria

**DOI:** 10.7759/cureus.1439

**Published:** 2017-07-07

**Authors:** Muhammad Junaid Ahsan, Rizwan Ishtiaq, Daniyal Ishtiaq

**Affiliations:** 1 Medicine, Interfaith Medical Centre; 2 Gastroenterology, Beth Israel Deaconess Medical Center, Boston; 3 General Medicine, Rawalpindi Medical College, Rawalpindi, Pakistan

**Keywords:** ischemic stroke, paroxysmal nocturnal hemoglobinuria, neutropenia

## Abstract

Paroxysmal nocturnal hemoglobinuria is a hematological disorder characterized by hemolytic anemia, cytopenia, and thrombotic events. Venous thrombotic events are more commonly reported. An arterial thrombosis is a rare event in paroxysmal nocturnal hemoglobinuria. We present a case of a 32-year-old female who had symptoms of stroke and on workup, she was diagnosed as a case of paroxysmal nocturnal hemoglobinuria.

## Introduction

Paroxysmal nocturnal hemoglobinuria (PNH) is characterized by a triad of complement-mediated hemolysis, cytopenia and venous thrombosis [1]. In paroxysmal nocturnal hemoglobinuria (PNH), a mutation of X-chromosomal phosphatidylinositol N-acetylglucosaminyl transferase subunit A (PIG-A) gene disorganizes the synthesis of glycosylphosphatidylinositol (GPI)-linked surface proteins which are the membrane inhibitors of CD59 and CD55 [2]. Common sites for the development of venous thromboembolism include liver, abdomen and the skin [3]. Thrombosis of the cerebral arteries in the patients with PNH is a rare entity and only a few cases have been reported in the literature to date. Informed consent statement was obtained for this study.

## Case presentation

**Figure 1:**
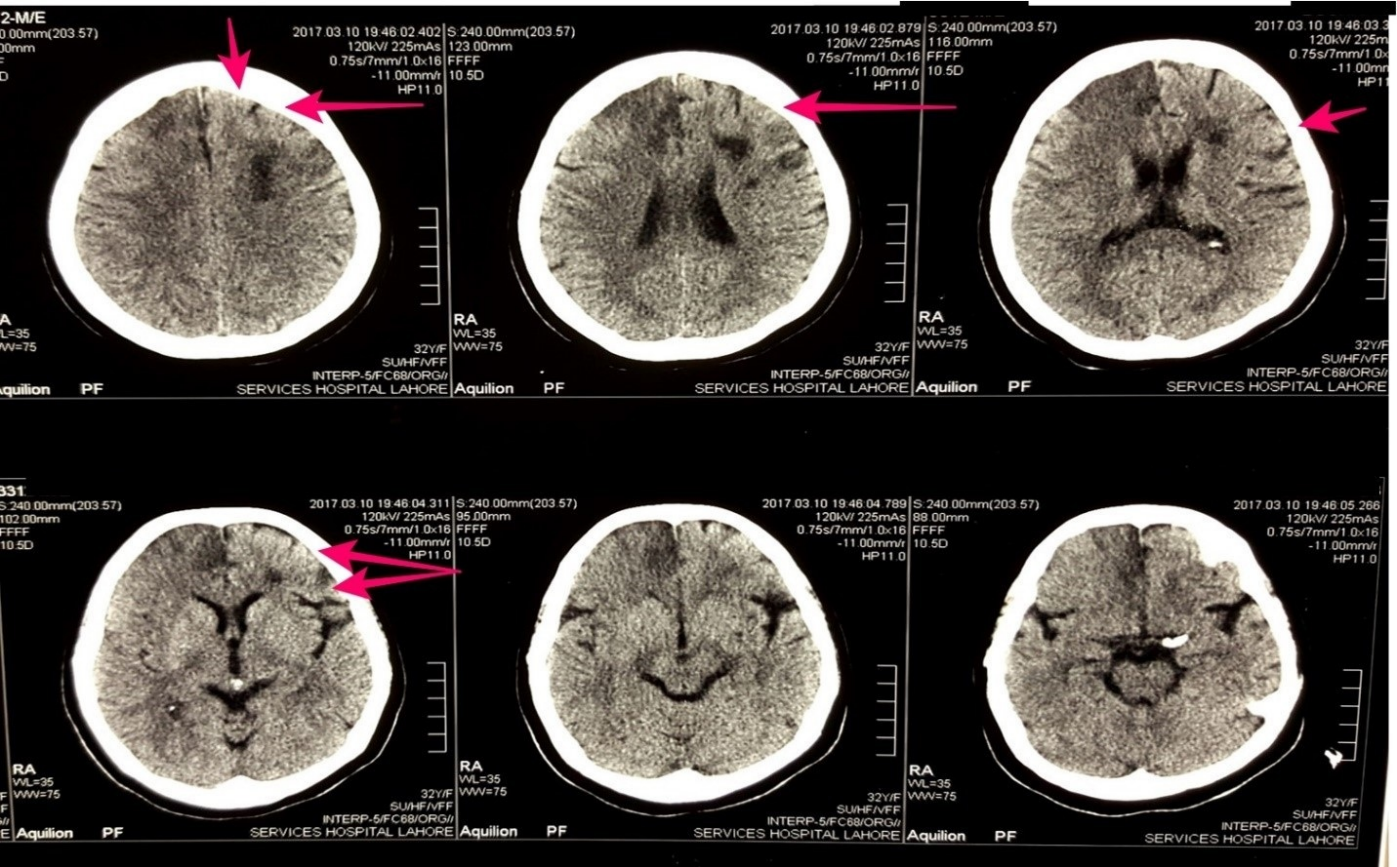
Figure showing the computed tomography scan of the patient without contrast. Red arrows show the hypodense areas in the right frontoparietal region

**Figure 2:**
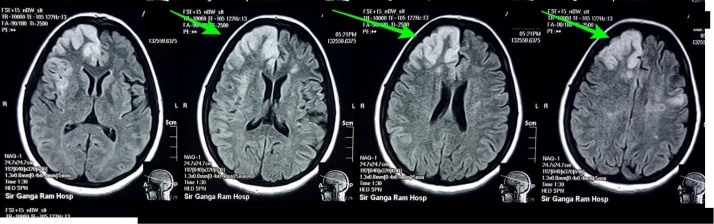
Figure showing the flair Images of the brain of the patient. Green arrows show infarct involving the right frontoparietal region of the brain

A 32-year-old normotensive, non-diabetic Asian female who was G5P3A2 presented to the emergency room with a one-day history of motor aphasia, weakness of left side of the body and two episodes of fits with an altered state of consciousness for the last 12 hours. Her vitals were normal with a blood pressure of 130/80 mmHg, a pulse of 80 beats per minute, afebrile and respiratory rate of 18/min. The patient looked pale and mildly icteric. Glasgow coma scale was 5/15. On physical examination, left plantar was up going and power was 2/5 in left upper and lower limb. Reflexes were brisk and hypertonia was noted. A non-contrast computed tomography (CT) scan was performed and a hypodense area in the right frontal region was found which suggested an infarction. Multiple hypodense areas in the right frontoparietal region were also seen. CT scan of the patient and flair images of the brain are shown in Figure 1-2.

Echocardiography showed 60% ejection fraction and carotid Doppler was normal. The patient was started on aspirin, atorvastatin, and warfarin. Foley catheter was passed and aspiration precautions and bed sore measures were instructed. On laboratory investigations, the patient had anemia with a hemoglobin of 7.5g/dL with the mean corpuscular volume of 106 fL, total leukocyte count of 3.2 and platelets was 241000/mm3. Lipid profile, liver function tests, and renal function tests were normal except a rise in indirect bilirubin levels. the patient's' stroke started to improve on the second day with patient becoming conscious on the third day of admission. The power of the left upper and lower limb returned to normal by the seventh day. During this time, the urine bag was found to have cola colored urine. Hemoglobin concentration dropped to 5.6 g/dL even after getting packed cell volume transfusions in that week. Based on raised bilirubin levels, blood in urine, pallor, and jaundice, the patient was thought to be having some sort of hemolytic anemia. There was no history of a sore throat, arthralgia, photosensitivity, alopecia and mouth ulceration ruling out systemic lupus erythematosus. Coombs test was performed which came out negative, lactate dehydrogenase (LDH) was 768 U/L, haptoglobin 12 mg/dL, reticulocyte count was 28 and coagulation profile was normal. The patient had a history of first two trimester abortions and further testing was performed. The patient tested negative for antinuclear antibodies (ANA), Anti-double-stranded deoxyribonucleic acid (Anti-dsDNA), Beta-2 microglobulin, and venereal disease research laboratory test (VDRL). Ham’s test was performed and the result came out positive. Flow cytometry was performed and below the normal values of CD55 and CD59 were recorded and the patient was diagnosed as a case of PNH with arterial thrombosis.

**Figure 3:**
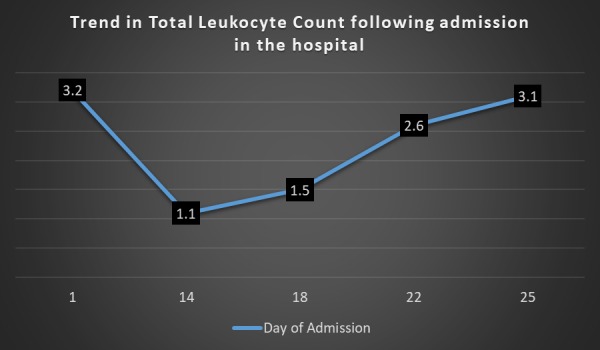
Figure showing the trends of neutrophils rise and fall of the patient

She was started on corticosteroids and tablet azathioprine 50 mg on the 10th day but on the 14th day, she started to get a high-grade fever with spikes ranging from 100-103F. Fever was continuous and not responsive to Panadol. Blood cultures came out negative. Chest X-ray, urine analysis, ultrasound abdomen, and pelvis were normal. On repeat complete blood count on the 14th day, the patient was found to have neutropenia along with anemia. Leukocyte count was 1.1 with an absolute neutrophilic count of 373/mm3. The patient was started on vancomycin B.D and piperacillin/tazobactam on eight hourly basis. By the 18th day, patient’s fever started to settle down and totally afebrile by the 20th day. Leukocyte count started to rise again on the 17th day and was 1.5 on the 18th day. It followed an increasing trend and became 2.6 on the 22nd day. Trends of neutrophils rise and fall are shown in Figure 3. Antibiotics were continued for 10 days and the patient was discharged on the 25th day after her leukocyte count became 3.1. The patient was discharged on azathioprine 50mg O.D, tablet folic acid, vitamin B12, and warfarin.

## Discussion

Several mechanisms have been proposed in the literature for thrombosis in patients with PNH. Hemolyzed red blood cells releasing thromboplastic material increased platelet activation in PNH patients and the procoagulant effect produced in the plasma by platelet-derived microparticles constitute these mechanisms [4]. The fact that the presentation of stroke during anemia supports the hypothesis that vascular accidents are linked with the hemolytic process.

Brubaker, et al. studied the life span of neutrophils in PNH patients [5]. They observed that the neutrophils lysis rate increased in PNH patients in comparison to controls when these cells were exposed to acidified fresh serum plus serum containing Anti-neutrophilic antibodies. Although the rate of lysis of neutrophils was increased, the intravascular life span of labeled neutrophils as measured by 32P-diisopropylfluorophosphate technique remained normal. This hypothesizes that the cause of neutropenia in patients with PNH is related to decreased production or increased lysis and not related to their life span.

Flow cytometry of the red blood cells to reveal decreased or reduced expression of CD55 and CD59 remains the gold standard test for PNH [6]. Treatment consists of heparin or low molecular weight heparin with long-term anticoagulation provided by warfarin [3]. In patients having venous thrombosis from PNH, lifetime anticoagulation is recommended [3]. Other medications include folic acid, iron replacement therapy, blood transfusion and corticosteroids [7]. Median survival time in patients with PNH is 10-15 years with most of the deaths occurring in the sixth decade of life, and thromboembolic complications constitute directly to the 60% of the deaths occurring in PNH [1,3].

Recently, there has been a lot of debate on the growing evidence of eculizumab which is a humanized monoclonal antibody. This drug binds to C5 inhibiting terminal complement system and blocking the formation of membrane attack complex [8]. Survival rate at five years in patients with PNH was 67% and with the use of eculizumab, this rate has improved to 96%. The rate of thrombotic events has also decreased with this medication. Previously, it was 6% per year and now it is less than 1% per year [7]. Eculizumab is an expensive medication and due to non-availability in our center, we were not able to use this medication for our patient. In cases of failed treatment or refractory to medication, a bone marrow transplant is an alternative option with long-term success [9]. Mortality related to bone marrow transplant is 42% [10].

For future considerations, a phase 2 clinical trial of the safety and efficacy of eculizumab in hemolytic PNH patients has been completed. Results have not been reported yet. Another phase 2 clinical trial is currently recruiting patients to study the efficacy of covers in PNH patients with resistance to eculizumab.

## Conclusions

Paroxysmal nocturnal hemoglobinuria (PNH) must be considered in the differential diagnosis of stroke in young children and adults with hemolysis. In previously undiagnosed patients with PNH, ischemic stroke can be the only initial presentation. PNH testing should also be made a part of thrombophilia screening as thrombophilia serves as an unrecognized cause of unprovoked thrombosis. Anticoagulation should be started urgently taking into consideration both long-term anticoagulation and bone marrow transplant. Better results can be produced with multidisciplinary involvement especially a hematologist.
